# Large-Scale Expansion of Human Liver Stem Cells Using Two Different Bioreactor Systems

**DOI:** 10.3390/bioengineering11070692

**Published:** 2024-07-09

**Authors:** Jan Thorbow, Andrea Strauch, Viktoria Pfening, Jan-Philip Klee, Patricia Brücher, Björn Boshof, Florian Petry, Peter Czermak, Maria Beatriz Herrera Sanchez, Denise Salzig

**Affiliations:** 1 Institute of Bioprocess Engineering and Pharmaceutical Technology, University of Applied Sciences Mittelhessen, 35390 Giessen, Germany; jan.barekzai@lse.thm.de (J.T.);; 2 Faculty of Biology and Chemistry, University of Giessen, 35392 Giessen, Germany; 3 2i3T, Società per la Gestione Dell’Incubatore di Imprese e per il Trasferimento Tecnologico, University of Turin, 10126 Turin, Italy

**Keywords:** stirred-tank bioreactor, multi-plate bioreactor, scale-up, oxygen limitation, *k_L_a*, long-term cultivation, human liver stem cells

## Abstract

The assessment of human liver stem cells (HLSCs) as cell therapeutics requires scalable, controlled expansion processes. We first focused on defining appropriate process parameters for HLSC expansion such as seeding density, use of antibiotics, optimal cell age and critical metabolite concentrations in conventional 2D culture systems. For scale-up, we transferred HLSC expansion to multi-plate and stirred-tank bioreactor systems to determine their limitations. A seeding density of 4000 cells cm^−2^ was needed for efficient expansion. Although growth was not significantly affected by antibiotics, the concentrations of lactate and ammonia were important. A maximum expansion capacity of at least 20 cumulative population doublings (cPDs) was observed, confirming HLSC growth, identity and functionality. For the expansion of HLSCs in the multi-plate bioreactor system Xpansion (XPN), the oxygen supply strategy was optimized due to a low *k_L_a* of 0.076 h^−1^. The XPN bioreactor yielded a final mean cell density of 94 ± 8 × 10^3^ cells cm^−2^, more than double that of the standard process in T-flasks. However, in the larger XPN50 device, HLSC density reached only 28 ± 0.9 × 10^3^ cells cm^−2^, while the glucose consumption rate increased 8-fold. In a fully-controlled 2 L stirred-tank bioreactor (STR), HLSCs expanded at a comparable rate to the T-flask and XPN50 processes in a homogeneous microenvironment using advanced process analytical technology. Ultimately, the scale-up of HLSCs was successful using two different bioreactor systems, resulting in sufficient numbers of viable, functional and undifferentiated HLSCs for therapeutic applications.

## 1. Introduction

Human liver stem cells (HLSCs) are multipotent stem cells that can be isolated from healthy liver donors [1]. They have been isolated from both primary cultures and cryopreserved human hepatocytes and have the potential to differentiate in vitro into a variety of cell types, including hepatocyte, endothelial, osteogenic and islet-like cells [1,2,3]. HLSCs can be expanded to yield large cell numbers while maintaining undifferentiated capabilities [1] and showing remarkable stability, as evidenced by the preserved telomere length during expansion [4]. HLSCs are negative for hematopoietic markers, human leukocyte antigen (HLA) class II and costimulatory molecules (CD40, CD80, CD86), but express some mesenchymal stromal cell (MSC) markers (CD90, CD105), albumin and α-fetoprotein, as well as cytokeratin (CK)-8 and CK-18. HLSCs also express conventional stem cell markers (vimentin and nestin) and embryonic stem cell markers (Oct3/4, Nanog, SSEA4, Sox2 and Musashi1), which are associated with self-renewal capacity and multipotency [2].

Preclinical studies showed that HLSCs are efficacious in a wide range of ischemic, degenerative, metabolic, inflammatory and autoimmune disease conditions [1,2,5,6,7,8]. In addition to their ability to repair, HLSCs also have many other potential therapeutic benefits due to their immunomodulatory effects [9]. Such effects have been attributed largely to their secretory products, such as immunoregulatory cytokines, growth factors and extracellular vesicles [10].

A recent phase I clinical trial in infants with inherited neonatal-onset hyperammonemia demonstrated the safety of HLSCs in clinical settings [8]. For this trial, HLSCs were isolated and expanded in a facility that complied with good manufacturing practices (GMP). A master cell bank (MCB) of HLSCs was established in 2011 (ML-011-01) from a donated liver fragment, following the standard criteria set by the Centro Nazionale Trapianti and the requirements of Directive 2001/20/EC [4,8]. Such clinical trials require large quantities of HLSCs and thus larger-scale manufacturing processes. However, it is unclear whether the HLSC immunophenotype and secretome differ according to the process scale, potentially the therapeutic application of these cells. There are few studies focusing on HLSC cultivation in bioreactors and in most cases the cells were differentiated into hepatocytes. HLSCs in a rotary bioreactor perfusion system lost their stem cell markers and acquired several markers that indicate hepatocyte commitment [11]. Bioreactor systems for the expansion of undifferentiated HLSC have not been described thus far. However, related cell types such as MSCs have been successfully expanded on a larger scale using various types of bioreactors, including hollow-fiber bioreactors [12,13], stirred-tank bioreactors (STRs) [14,15,16,17,18], vertical-wheel bioreactors [19] and multi-plate bioreactors [20]. Given that HLSCs and MSCs share some characteristics (e.g., phenotype, surface markers CD90 and CD105), the bioreactor types and process strategies used for MSC expansion and scale-up should be adaptable for HLSCs.

In the current study, we focus on multi-plate bioreactors (10 and 50 plates) and an STR (2 L glass vessel equipped with a marine impeller) to establish a process for HLSC expansion. These systems differ in geometry and the hydrodynamic forces applied to the cells, and therefore require different process strategies. Multi-plate bioreactors have been used to cultivate human periosteum-derived stem cells [20] and hepatic progenitors [21]. MSCs have also been expanded in this type of bioreactor for applications such as bone regeneration and osteoarthritis treatment [22]. In the multi-plate bioreactor, cells are seeded on a static tissue-culture plastic surface, similar to standard T-flasks. Moreover, process parameters such as dissolved oxygen concentration (DO) and pH can be monitored in-line to improve control over the expansion process. Although HLSCs have never been expanded in an STR, this is the most commonly used bioreactor type for MSC expansion [23]. Typically, microcarriers are used in STRs to provide a growth surface for the cells, and several microcarrier types have already been tested with MSCs [24]. STRs offer not only in-line monitoring but also control of process parameters such as DO, pH and temperature, enabling advanced monitoring and control of parameters like cell growth or metabolite concentrations. Therefore, the STR appears to be a promising system for HLSC expansion and was selected for testing in this study.

The aim of the study was to establish a scalable and controlled process for HLSC expansion and to compare two different bioreactor systems. Initially, we established baseline attachment and growth characteristics in T-flasks, evaluating process characteristics and quality attributes such as cell viability, identity and functionality. We also investigated the effect of seeding density, toxic metabolites, antibiotics and the effect of cell aging on HLSCs. In the next phase, we investigated the transfer of HLSC expansion to the multi-plate bioreactor system Xpansion. We investigated oxygen transfer within the system and examined HLSC expansion at two different scales: the 10-plate (XPN10) and 50-plate (XPN50) devices. We screened various microcarriers in 100 mL spinner flasks and established an HLSC expansion process in a 2 L bioreactor with advanced online monitoring of process parameters.

## 2. Materials and Methods

### 2.1. Cultivation of HLSCs in T-Flasks

The HLSCs (ML-011-01, courtesy of Unicyte AG, Oberdorf, Switzerland) were isolated as previously described [8]. For routine culture, the cells were seeded in αMEM supplemented with 10% (*v*/*v*) FBS, 2 mM GlutaMax (both from Thermo Fisher Scientific, Darmstadt, Germany), 4 ng mL^−1^ FGF-2 and 4 ng mL^−1^ EGF (both from Miltenyi Biotec, Bergisch Gladbach, Germany) (growth medium) at a density of 4000 cells cm^−2^ in T-75 or T-175 flasks (Sarstedt, Nümbrecht, Germany) at 37 °C, in a 5% CO_2_ atmosphere. The medium was replaced after 24 h. Once the cells reached 85 ± 5% confluence (usually after 120 h), they were detached using TrypLE (Gibco, Schwerte, Germany) for 5 min at 37 °C. Unless otherwise stated, the cells were grown without antibiotics.

### 2.2. Cultivation of HLSCs in Hyperflasks

The cells were seeded at a density of 4000 cells cm^−2^ in growth medium using Hyperflasks (Corning, New York, NY, USA) and were incubated at 37 °C, 5% CO_2_. At 85 ± 5% confluence, the cells were detached using TrypLE for 5 min at 37 °C.

### 2.3. Investigation of HLSC Expansion in the Xpansion Bioreactor Systems

#### 2.3.1. Setup of the XPN Devices and HLSC Expansion

The XPN devices (XPNBRS; Pall, New York, NY, USA) were set up according to the manufacturer’s recommendations. To cultivate HLSCs in the XPN10 bioreactor, we prepared 1.6 L of growth medium in a sterile 2 L medium bottle (Pall). For the XPN50 bioreactor, we prepared 5.8 L of growth medium in a 10 L pooling bottle (Pall). Prior to inoculation, each device and the growth medium were preheated for 24 h at 37 °C in an incubator. XPN10 parameters were PID-controlled and set as follows: temperature 37 °C, 20 mL min^−1^ air, 30% DO, pH 7.3 and stirrer speed 23 rpm. The same parameters were used for the XPN50, except the stirrer speed was increased to 34 rpm. The devices were inoculated with 4 × 10³ cells cm^−^² and the following intermittent stirring was applied: 3 min of stirring at 23/34 rpm (to ensure proper cell distribution within the reactor), followed by 2.5 h without stirring for attachment, another 3 min of stirring at 23/34 rpm and subsequent 2.5 h without stirring. Samples from the supernatant were taken daily for metabolite analysis (Section 2.5.4). After 4–5 days, the cells were detached using a 1:2 diluted TrypLE/PBS solution (Biochrom, Berlin, Germany) for 20 min with four shaking cycles (each 10 shakes) on the harvest station (Pall). The cells were then counted (Section 2.5.1) and their identity (Section 2.5.5) and functionality (Section 2.5.6) were confirmed.

#### 2.3.2. Determination of the Oxygen Uptake Rate (*OUR*)

The *OUR* was determined using the dynamic method [25] while culturing HLSCs in the XPN10. Briefly, oxygen gassing was turned off for 20–30 min and then turned on again. The DO was monitored online. The *OUR* was calculated using Equation (1):

(1) $$OUR=-r_{O2}\times X=\frac{c_{i}-c_{i-1}}{t_{i}-t_{i-1}}$$

where *r_O_*_2_ is the oxygen consumption rate [mol (h cell)^−1^], *X* is the cell number [cell] and *c* is the oxygen concentration at time *t* [mol].

#### 2.3.3. *k_L_a* Measurement

The XPN10 was prepared as described in Section 2.3.1 and filled with 1.6 L of water instead of cells and medium. It was then placed in an incubator, and the temperature of the water was monitored until it stabilized at 37 °C, marking the start of the *k_L_a* measurement. The XPN10 was aerated with 60 mL min^−1^ air and stirred at 150 rpm to achieve the maximum DO concentration (c*), which was set as 100%. The air gassing was then stopped, and 50 mL min^−1^ of N_2_ was used to replace the DO. When the DO level reached 15%, the process was stopped, and the system was re-gassed with air/pure oxygen. The stirring speed was set to 23 rpm and the air/oxygen input to 40 mL min^−1^ to simulate the cultivation parameters used for expanding HLSCs in the XPN10 bioreactor. The *k_L_a* was calculated using Equation (2):

(2) $$k_{L}a=\frac{ln\left(\frac{\bar{c}-c_{i-1}}{\bar{c}-c_{i}}\right)}{t_{i}-t_{i-1}}$$

where $\bar{c}$ is the saturated DO [mol] and *c* is the DO at time *t* [mol].

### 2.4. Investigation of HLSC Expansion in the Stirred-Tank Bioreactor Systems

#### 2.4.1. Screening in Spinner Flasks

The cells were cultured in 100 mL spinner flasks containing 50 mL of growth medium at a seeding density of 4000 cells cm^−2^. The stirring speed was initially set to 27 rpm, then increased to 40 rpm after 48 h, and to 50 rpm after 72 h to prevent agglomeration. We tested various microcarriers: Cytodex I, Star Plus (both from Cytiva, formerly GE Healthcare, Solingen, Germany), Synthemax II and Enhanced Attachment (both from Corning). The spinner flasks were incubated at 37 °C in a 5% CO_2_ atmosphere. Samples were taken daily for the analysis of cell density and microscopic examination of growth and cell distribution, following staining with SYBR-Green [26]. The HLSCs were detached using TrypLE and counted (Section 2.5.1) or the cell number was determined by nuclear staining (Section 2.5.2).

#### 2.4.2. Setup and HLSC Expansion Using a STR

HLSCs were expanded in a 2-L glass STR (Labfors 5 Cell System; Infors, Bottmingen, Switzerland) with a working volume of 1 L growth medium. The temperature was set to 37 °C, the DO to 40% (controlled by air/oxygen supply over the headspace) and the pH to 7.3 (controlled with 1 M sodium hydroxide and CO_2_ flow). The reactor was equipped with online probes for temperature, pH, DO (PID controllers) and dielectric spectroscopy (for in-line cell growth analysis) to allow comprehensive process monitoring and control. The microcarrier growth surface consisted of 3 g L^−1^ Cytodex I. The STR was inoculated at a density of 4000 cells cm^−2^. The stirrer speed was initially set to 30 rpm and increased to 40 rpm after 72 h. Daily samples were taken to determine cell numbers (Section 2.5.2), cell distribution on the microcarrier (Section 2.5.3) and metabolite concentrations (Section 2.5.4). After 5–6 days, the cells were detached using TrypLE as described above. The cells were counted (Section 2.5.1), and their identity (Section 2.5.5) and functionality (Section 2.5.6) were confirmed.

### 2.5. HLSC Analytics

#### 2.5.1. Cell Count, Doubling Time and Viability based on Trypan Blue Staining

Cell viability was assessed by membrane exclusion [27]. Briefly, detached cells were treated with 0.5% (*w*/*v*) trypan blue and counted using a hemocytometer (Neubauer counting chamber) to distinguish between living and dead cells. A minimum of 100 cells was counted per square. The cell concentration was calculated using Equation (3):

(3) $$Cell\;concentration\;\left[\frac{cells}{mL}\right]=\frac{dilution\times chamberfactor\times counted\;cells}{number\;of\;squares\;counted}$$

with a chamber factor (Neubauer counting chamber) of 10^4^.

The doubling time was calculated using Equation (4):

(4) $$Doubling\;time\;\left[h\right]\;t_{D}=\frac{ln\left(2\right)\;}{ln(X_{i}/X_{i-1})/\left(t_{i}-t_{i-1}\right)}$$

The viability [%] was calculated using Equation (5):

(5) $$Viability\;\left[\%\right]=\frac{living\;cells\;}{living\;cells+dead\;cells}\times 100\%$$

#### 2.5.2. Cell Count-Based Crystal Violet Staining

Cell nuclei were counted as previously described [28]. Briefly, samples of the microcarriers were collected from the STR and incubated with an equal volume of 0.2% (*w*/*v*) crystal violet in 0.2 M citric acid at 37 °C for 60 min. At least 100 nuclei per square were counted using a hemocytometer. The cell concentration was then calculated using Equation (3), with the total number of nuclei replacing the total number of cells.

#### 2.5.3. Cell Distribution on the Microcarriers

The nuclei of HLSCs on microcarriers were stained using SYBR-Green as previously described [24]. Briefly, the cells on the microcarrier were fixed with 4% paraformaldehyde (Merck, Darmstadt, Germany) for 15 min at room temperature and stained with SYBR-Green solution for 5 min at room temperature in the dark. Each sample was then washed twice with PBS before imaging with a Cytation 3 (BioTek Instruments, Winooski, VT, USA).

#### 2.5.4. Metabolic Analysis

Glucose and lactate concentrations were determined using a Biosen C-line analyzer (EKF Diagnostic, Barleben, Germany) whereas ammonia concentrations and lactate dehydrogenase (LDH) activity were measured using a Cedex Bio Analyzer (Roche, Basel, Switzerland). The cell-specific conversion rates *q* [g cell^−1^ h^−1^] were calculated using Equations (6) and (7):

(6) $$\mathrm{Metabolic}\;\mathrm{conversation}\;\mathrm{rate}\;[g\;{\mathrm{cell}}^{-1}\;h^{-1}]\;q=\frac{\left(c_{i}-c_{i-1}\right)\times V}{\bar{x}\times \left(t_{i}-t_{i-1}\right)}$$

with

(7) $$\mathrm{Logarithmical}\;\mathrm{average}\;\mathrm{cell}\;\mathrm{count}\;\bar{x}=\frac{x_{i}-x_{i-1}}{ln(x_{i})-ln\left(x_{i-1}\right)}\;$$

where *c* is the metabolite concentration [mol] and *V* is the working volume at time *t * [L].

#### 2.5.5. HLSC Identity (CD Marker Determination)

HLSCs were characterized by flow cytometry using a panel of antibodies targeting cell surface markers (CD105, CD73, CD90, CD29, CD14, CD34 and CD45) and intracellular albumin (Table S1). Antibodies conjugated to FITC, PE or APC were used for each marker, along with isotype controls where applicable. A Guava easyCyte flow cytometer (6HT-2L; Merck) was used to determine the percentage of cells positive for each marker and HLSC identity.

#### 2.5.6. HLSC Functionality (Islet Formation Assay)

We carefully transferred 7 × 10^7^ HLSCs into a Hyperflask containing 560 mL of RFG medium (RPMI 1640 supplemented with 10% FBS and 11.6 mM glucose [3]. To initiate differentiation, we added 1.12 mL of protamine stock solution (10 mg mL^−1^) to give a final concentration of 20 mg mL^−1^. The cell suspension was gently mixed by hand to ensure homogeneity and any air bubbles were meticulously removed using a pipette. The Hyperflask was then gently shaken to achieve a good distribution of cells. The cells were incubated at 37 °C in a 5% CO_2_ atmosphere for 7 days. During the first 4 days after seeding, the cells were left undisturbed to facilitate spheroid formation. HLSC islets (HLSC-ILS) typically formed within the first 12–48 h and retained their 3D spheroidal morphology throughout the 7-day incubation period, as long as they remained in the same Hyperflask in which they differentiated and the medium was not changed. On day 7, the HLSC-ILS were manually collected from the Hyperflasks, centrifuged (300 g, 5 min, room temperature) and gently re-suspended to obtain a homogeneous suspension. They were then counted, and viability was evaluated using fluorescein diacetate (FDA; Thermo Fisher Scientific) and propidium iodide (PI; Sigma-Aldrich, Taufkirchen, Germany).

## 3. Results

### 3.1. Baseline Experiments for Scale-Up Using Static T-Flasks

#### 3.1.1. HLSCs Attached Firmly within 3 h

To minimize the presence of non-attached HLSCs, it is necessary to understand their attachment kinetics. Therefore, we examined the time required for HLSCs to adhere to TC-treated polystyrene surfaces in T-flasks. We found that the HLSCs had already adhered to the surface within 2 h and begun spreading (Figure S1). After 3 h, all cells had firmly attached to the surface, and even gentle shaking did not dislodge them.

#### 3.1.2. A Seeding Density of 4 × 10^3^ cell cm^−2^ without Antibiotics Was Optimal for HLSC Growth

We conducted tests to determine the minimum seeding density required for sufficient cell growth, aiming to minimize the number of seeded cells while achieving maximum expansion per cell for efficient and cost-effective scale-up. HLSCs were seeded at densities ranging from 1–10 × 10^3^ cells cm^−2^, and we analyzed their growth kinetics over 120 h. The highest maximum cell density (38 × 10^3^ cell cm^−2^) and expansion factor (9.5) were observed when cells were seeded at 4 × 10^3^ cells cm^−2^ (Figure S2). We therefore maintained a seeding density of 4 × 10^3^ cells cm^−2^ for subsequent experiments unless otherwise specified.

Next, we examined the effect of antibiotics on HLSC growth. Although antibiotics are often added to cell cultures, they can significantly impair growth [29] and they should not be needed if sterility is ensured during all process steps. We found that penicillin/streptomycin slightly reduced the growth rate of HLSCs (*µ_Antibiotics_* = 0.031 ± 0.002 h^−1^, *µ_Control_* = 0.037 ± 0.004 h^−1^). However, antibiotics had no impact on the overall expansion factor or maximum cell density after 144 h (Figure S3).

#### 3.1.3. HLSCs Can Grow over an Extended Number of Cumulative Population Doublings

**Figure 1:**
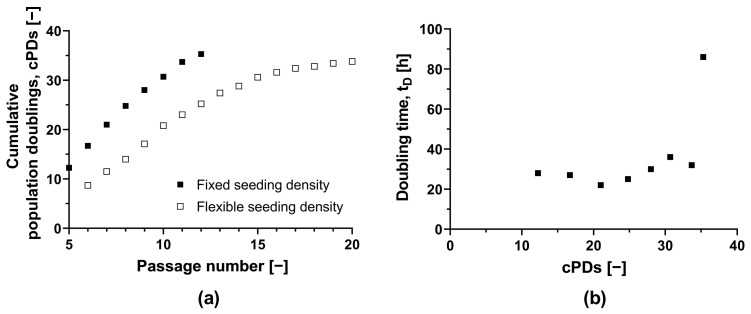
Long-term culture of HLSCs. (**a**) Absolute cumulative population doubling of HLSCs plotted against passage number. (**b**) Doubling time plotted against cumulative population doublings of HLSCs. HLSCs were split and seeded with a fixed seeding density of 3 × 10^3^ cells cm^−2^ (black squares) and a flexible seeding density (white squares) of 2–14 × 10^3^ cells cm^−2^. HLSCs were cultured in αMEM growth medium at 37 °C and a 5% CO_2_ humidified atmosphere for 120 h.

To assess the feasibility of a scale-up process, we determined the maximum number of cPDs that maintained a sufficient HLSC growth rate and a stable phenotype (Figure 1a). Starting with the initial MCB (ML-011-01, cPD 7.9), we observed that the doubling time in T-175 flasks remained adequate for up to an additional 22.8 PDs, despite increasing from 22 h (equivalent to 21 cPDs) to 36 h (equivalent to 30.7 cPDs) (Figure 1b). Subsequent expansion led to a further increase in doubling time to 86 h. HLSC growth ceased completely after 35.3 cPDs. In a second independent experiment with an alternate passaging strategy, we achieved a similar maximum number of 33.8 cPDs (Figure 1). The natural limit for in vitro HLSC growth is thus ~34 cPDs, including isolation, storage as master and working cell banks, and any subsequent expansion.

After 30.7 cPDs, morphological abnormalities were observed, including elongation (Figure S4). Additionally, the CD105 surface marker signal fell below 85% after 30.7 cPDs, suggesting the HLSC-specific phenotype was lost (Table S2). However, we confirmed that the HLSCs remained functional after 20.5 cPDs, as evidenced by their ability to form viable islet-like structures (64.8 ± 5.7%), yielding 27.6 IEQs (islet equivalents) per 100 ILS. Therefore, HLSCs from this specific donor can be expanded safely for 20 cPDs without losing functionality.

#### 3.1.4. Lactate and Ammonia Significantly Inhibit HLSC Growth

Lactate and ammonia are toxic metabolites [30], so it is necessary to determine critical concentrations that could inhibit HLSC growth. We defined growth inhibition as a decrease of more than 30% compared to the control. We observed 27% growth inhibition in the presence of 10 mM lactate, indicating a critical lactate concentration of 10–20 mM (Table 1). Furthermore, HLSC growth was inhibited by 70% in the presence of 2 mM ammonia, indicating a critical ammonia concentration of 1–2 mM (Table 2). However, lower concentrations of both metabolites significantly reduced HLSC growth.

### 3.2. Scale-Up of HLSC Expansion Using the Xpansion Bioreactor System

We compared two different bioreactor systems for the large-scale expansion of HLSCs, starting with the multi-plate Xpansion bioreactor system as 10-plate (XPN10) and 50-plate (XPN50) devices.

#### 3.2.1. XPN10 Has a Low *k_L_a* and Supplementary Oxygen Is Required for HLSC Expansion

In preliminary experiments, we identified the oxygen supply as a critical process parameter for the XPN10 bioreactor. The system-specific *k_L_a* for this device was 0.028 h^−1^, a much lower value compared to other systems. For example, the *k_L_a* for a 96-well plate is 20–250 h^−1^, whereas that of a shaking flask is 10–180 h^−1^ [31]. To determine whether the oxygen transfer rate (*OTR*) satisfied the *OUR*, we measured the *OUR* of the HLSCs in the XPN10 at a confluent state (101,307 cells cm^−2^). The *OUR* was 3.04 × 10^−5^ mol h^−1^, corresponding to a cell-specific oxygen respiration rate (*rO*_2_) of 0.49 × 10^−13^ mol h^−1^ cell^−1^. Interestingly, the *rO*_2_ of HLSCs grown in T-75 flasks was 3-fold higher (1.79 × 10^−13^ mol h^−1^ cell^−1^). We then calculated the oxygen supply in the XPN10 to see if it was adequate for HLSC expansion. Considering a high cell density (6.2 × 10^8^ cells for the 6120 cm^2^ growth surface of the XPN10), the minimal required *k_L_a* was 0.076 h^−1^ for aeration with air and 0.019 h^−1^ for aeration with pure oxygen. Thus, only pure oxygen aeration could ensure a sufficient oxygen supply. This was confirmed by preliminary experiments showing that the cell density in the XPN10 with air aeration was 2.6-fold lower compared to runs with pure oxygen aeration. Moreover, the doubling time was 60% higher in the air-aerated runs, resulting in 1.9-fold fewer population doublings.

#### 3.2.2. Scale-Up from T-Flask to the XPN10 Was Successful

Based on the findings reported in Section 3.1, the XPN10 was inoculated with 4 × 10^3^ cells cm^−2^ using growth medium without antibiotics. We allowed the optimal attachment time (Section 3.1.1) followed by an intermittent stirring, inoculation and attachment strategy: 3 min stirring at 23 rpm (to ensure proper cell distribution within the reactor), followed by 2.5 h without stirring for attachment, another 3 min of stirring at 23 rpm and another 2.5 h without stirring. After 24 h, all cells had either attached or lysed following apoptosis (none were found in the supernatant). Nevertheless, population doublings were calculated assuming that all cells had completely attached to the XPN10 growth surface. The calculated cPD is therefore underestimated, because fewer cells attaching during the early phase would result in an even higher cPD.

In three independent runs, cells reached confluence after 120 h in culture (observed by microscopy) and were then detached by incubating with 2-fold diluted TrypLE at room temperature for 20 min, based on preliminary experiments (Table S3). This achieved high cell densities at harvest, minimized agglomeration and reduced costs by saving on materials. A final mean cell density of 94 ± 8 × 10^3^ cells cm^−2^ was achieved with 4.1 ± 0.3 cPDs and a doubling time of 30 ± 2 h (Table 3). In comparison, the control (HLSC expansion in static T-flasks) resulted in cell densities of 44 ± 2 × 10^3^ cells cm^−2^ and a cPD of 3.5 ± 0.1. We harvested a total of 5.8 ± 0.5 × 10^8^ cells from one XPN10 bioreactor.

**Figure 2:**
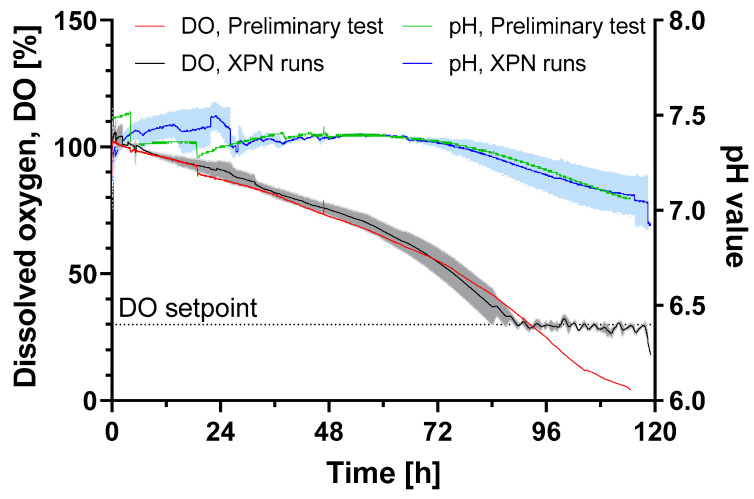
DO and pH value of HLSC expansion in an XPN10 bioreactor in αMEM growth medium at 37 °C and a 5% CO_2_ humidified atmosphere for 120 h. The XPN10 was inoculated with 4 × 10^3^ cells cm^−2^ and aerated with air (preliminary test, n = 1) or pure oxygen (runs, n = 3) while the pH was monitored but not controlled. Data are means (solid line, n = 3) ± SD (colored background).

All surface markers (CD105, CD73 and CD29) were present on HLSCs expanded in the XPN10. A glucose consumption rate of 1.8 ± 0.1 × 10^−11^ g cell^−1^ h^−1^ was observed, which was 3-fold higher compared to human periosteum-derived stem cells expanded in an XPN [20]. However, glucose limitation did not occur until the end of the cultivation period. The oxygen supply was also sufficient throughout the cultivation, and the pH remained above the critical value of 7 (Figure 2).

#### 3.2.3. Process Transfer to the Larger XPN50 Bioreactor Needs Further Optimization

Oxygen transfer was a critical process parameter in the XPN10, so careful consideration of the oxygen supply was necessary for process scale-up to the XPN50. According to Fick’s first law, there are two avenues to increase the OUR in the cell culture medium: first by increasing the concentration profile Δ*c* (difference between oxygen concentration inside the tube and outside) and second by reducing the diffusion distance Δ*x*. By introducing pure oxygen instead of air, the Δ*c* increased ~5-fold. To shorten the diffusion distance Δ*x*, the laminar boundary layer around the tube in the center of the XPN10 can be reduced by increasing the power input, which in this case required a higher stirring speed. The stirrer speed was therefore integrated into the oxygen control loop, and the following aeration strategy was implemented to optimize oxygen transport during cultivation: if DO fell below 30%, the air flow was increased, oxygen flow was initiated, the oxygen flow was increased and the stirrer speed was increased.

Using this scale-up strategy, we successfully expanded the HLSCs to the XPN50 reactor. However, the final HLSC density after 120 h was only 28 ± 0.9 10^3^ cells cm^−2^. Despite efforts to reduce shear stress during the inoculation, introducing washing steps or different strategies for pre-cultures (Hyperflask and T-flask), this value remained unchanged. Notably, a higher glucose consumption rate of 8.2 ± 1.3 10^−11^ g cell^−1^ h^−1^ was observed compared to the XPN10. The accumulation of lactate (<8 mM) and ammonia (<1 mM) did not reach growth-inhibitory levels (Section 3.1.4) and was unlikely to explain the low harvesting density, particularly when compared to the initial XPN10 data (Table 3). Nevertheless, all identity markers were confirmed (>99% for CD29, CD73 and CD105, and >80% for CD90) and HLSCs remained functional after expansion in the XPN50, as evidenced by their ability to form viable islet-like structures (63.5 ± 3.3%) yielding 39 IEQs per 100 ILS. Although the XPN50 bioreactor provides an attractive opportunity to expand HLSCs at a larger scale with lower shear forces and controlled conditions, its limitations in terms of cell yield at this scale mean that significant process development is required for further scale-up when using HLSCs with the XPN system.

### 3.3. Scale Up of HLSC Expansion Using a STR

Given that the oxygen supply is critical for effective scale-up, we tested the STR as a second bioreactor system because of its higher *k_L_a* (1-L STR, *k_L_a* = 60–360 h^−1^ [31]) and its common use for related cell types such as MSCs.

#### 3.3.1. Cytodex 1 Microcarriers Were Suitable for HLSC Attachment and Growth

The expansion of strictly adherent HLSCs in an STR requires the use of microcarriers. We therefore screened four different microcarriers in small-scale spinner flasks. Star Plus and Synthemax II promoted cell aggregation, resulting in low attachment rates. In contrast, Cytodex 1 and Enhanced Attachment microcarriers ensured sufficient cell attachment. However, significant cell growth was only achieved when using the Cytodex 1 microcarriers, yielding 16 × 10³ cells cm^−^². Cytodex I microcarriers were therefore used for all subsequent investigations in the STR.

#### 3.3.2. HLSC Expansion Can Be Achieved Using a 1-L STR

In the three STR runs, we observed a 24 h lag phase followed by an exponential growth phase. Typically, HLSCs reached the stationary phase after 120–144 h. The presence of HLSC on Cytodex 1 microcarriers was confirmed by microscopic examination (Figure S5) prior to cell harvest. Final cell densities and yields, population doublings and doubling times are shown in Table 4. Growth characteristics varied among the runs, mainly due to differences in the pre-cultures and/or handling issues. Cell density was monitored online using a dielectric spectroscopy probe [32] (Figure S6a). This revealed a high degree of correlation with offline cell counts, thus indicating similar growth rates (*µ_online_* = 0.031 h^−1^; *µ_offline_* = 0.028 h^−1^).

Glucose and lactate concentrations were also determined (Figure S6b). In runs 1 and 3, glucose limitation was observed after 120 h, prompting the addition of glucose at that point. In run 2, glucose limitation was observed after only 72 h, so the glucose bolus was added earlier. However, the glucose consumption rate during the exponential growth phase was similar in all STR runs (7.0 ± 4.7 × 10^−13^ mol h^−1^ cell^−1^), as was the lactate concentration at the end of the cultivation period (12.6 ± 1.7 mM). The ammonia concentration (1.0 ± 0.1 mM) was below the levels that would inhibit cell growth. We also measured enzyme lactate dehydrogenase (LDH) as an indicator of cell damage [33]. A significant increase in LDH was observed during run 1, but no comparable increase was detected in the other two runs. The identity of the HLSCs expanded in the STR was verified, showing CD90 and CD105 marker expression at ≥90%, and CD73 at least at 85%. The functionality of the expanded cells was confirmed by their ability to form viable islet-like structures (70.1 ± 11.9%), yielding 39 IEQs per 100 ILS.

## 4. Discussion

Large-scale clinical applications require significant numbers of HLSCs, which are difficult to produce using traditional culture methods. We evaluated two bioreactor systems capable of expanding HLSCs—multi-plate bioreactors at two scales and a 2-L STR—both of which offer advantages of scalability, a controlled microenvironment (Table S4), GMP compliance and maintenance of cell functionality.

The transition from T-flasks to the multi-plate bioreactors was straightforward, requiring only minor modifications to the T-flask culture process. We systematically developed an optimal seeding and harvesting strategy for HLSCs, observing only slight effects from antibiotics, but growth was inhibited by high concentrations of lactate and ammonia. HLSCs exhibited similar ammonia tolerance to MSCs but lower lactate tolerance. Specifically, MSC growth was inhibited at a lactate concentration of 35.4 mM and an ammonia concentration of 2.4 mM [30]. This suggests that efficient mixing is necessary during scale-up to avoid locally high concentrations of ammonia or lactate. Additionally, constant monitoring of ammonia and lactate concentrations is essential for successful HLSC expansion.

Scaling up from the XPN10 to the XPN50 bioreactor resulted in growth comparable to that in the T-flask, but significantly reduced growth compared to the XPN10. Despite the similar conditions, HLSCs showed reduced growth in the larger bioreactor. This difference may reflect variations in fluid dynamics and bioreactor-specific features and handling. Notably, the XPN10 has a lower surface area-to-volume ratio (3.8 cm^−1^) than the XPN50 (5.4 cm^−1^) so less medium, nutrients and oxygen are available per cell in the XPN50, leading to higher concentrations of toxic metabolites. Although lactate and ammonia levels were within acceptable limits, mammalian cell metabolism is complex and may produce additional potentially toxic metabolites that were not analyzed in this study. Altered microenvironments may prompt metabolic shifts in HLSCs. Indeed, we observed an 8-fold increase in glucose consumption correlated with reduced growth, indicating metabolic adaptation and suggesting inefficient anaerobic energy production despite non-critical oxygen levels. This observation contrasts with other studies that did not report significant changes in glucose consumption during XPN scale-up [20]. Handling differs significantly between the XPN10 and XPN50. The smaller scale requires ~1.6 L of medium and cell suspension to be pumped into the bioreactor, whereas the XPN50 requires more than three times this volume (5.6 L). Consequently, longer pumping times and transport distances are needed to distribute HLSCs throughout the bioreactor, exposing them to higher shear forces (in the tubing and within the bioreactor) and an unregulated system with fluctuating temperatures, leading to corresponding pH fluctuations. These effects were also evident during HLSC harvest and may explain the reduced cell yield.

The STR facilitated HLSC expansion comparable to the T-flask and XPN50 systems but significantly lower than the XPN10. Despite their reliance on fluid movement, the nature of this movement differs significantly between the systems. The XPN system circulates media with laminar flow, whereas the STR creates more turbulence. The latter offers benefits such as a homogeneous environment throughout the reactor but requires careful control of the power input to avoid excessive shear stress. Even with minimal power input to ensure full microcarrier suspension [34], the shear stress may have exceeded the tolerance of HLSCs, inhibiting their expansion. Sources of shear stress include turbulent flow, particle collisions and collisions with reactor walls, baffles and probes, which may explain the reduced growth rates we observed. Such effects require careful evaluation, especially for sensitive mammalian cells, and should be correlated with the critical quality attributes of the final HLSC product.

Another difference between the XPN bioreactors and STR is the structure and material of the growth surfaces. The surface material can significantly affect MSC growth [35,36]. Whereas the T-flasks and XPN bioreactors are made of polystyrene, the STR contains dextran-based microcarriers with a microporous structure. Although these microcarriers were selected by screening for their ability to support HLSC attachment and expansion, this might not necessarily yield maximum growth. In fact, the suitability of Cytodex 1 microcarriers for cell expansion has been previously reported, some studies indicated limited growth [26,37]. Custom-designed microcarriers with adaptations such as biological coatings or increased porosity might offer a solution for shear-sensitive cells.

However, from a scale-up perspective, the XPN system presents two clear limitations: (i) limited scalability (the current setup allows for up to 200 culture plates with essentially the same geometry), and (ii) oxygen limitations during scale-up. While the development of a control loop including gas flow, gas mixture and stirring speed was sufficient for XPN10 and XPN50, it will be challenging to ensure a sufficient oxygen supply at larger scales because there are no remaining options for increasing the oxygen transfer rate without altering major raw materials such as the medium composition. Despite these limitations, the XPN system remains valuable for HLSC expansion due to its promising performance in smaller applications. The STR offers clear advantages for HLSC expansion by providing robust control of oxygen and glucose limitations. With advanced online cell monitoring capabilities, the STR allows the direct control of HLSC growth. Further development and a deeper understanding of the cellular responses to higher shear forces in the STR will be required to achieve higher cell densities.

## 5. Conclusions

This study demonstrated the scalability of HLSC expansion in two distinct bioreactor systems: the XPN and STR. Regardless of the growth surface or hydrodynamic conditions in the bioreactors, all cultivations produced viable HLSCs in terms of identity and functionality. The successful expansion of HLSCs can be attributed to the control of critical process parameters. Both bioreactor systems provide control of the dissolved oxygen, making them suitable platforms for scalable HLSC expansion. The XPN system yielded higher quantities of HLSCs, but its scalability is limited to a 20-fold increase in surface area (XPN200). Additionally, even at the XPN50 scale, the need for additional equipment and human resources increased the scale-up costs. On the other hand, STRs offer a high surface-to-volume ratio and can be scaled up to 2000 L, making them a more cost-effective solution. However, the process setup for STRs is more complex, requiring comprehensive investigation and a thorough understanding of the process. Notably, the impact of hydrodynamics on HLSCs has yet to be investigated. Even so, the scale-up of HLSCs was achieved using two very different bioreactor systems, resulting in a sufficient number of viable, functional and undifferentiated HLSCs.

## Figures and Tables

**Table 1:** The effect of lactate added at the start of cultivation on the growth of HLSCs over 120 h. Growth inhibition was defined as a reduction in cell density and population doublings of less than 70% of the control flask (0 mM lactate). Data are means ± SD (n = 3).

Added Lactate Concentration [mM]	Cell Density [×10^3^ Cells cm^−2^]	Population Doublings [−]	Growth Inhibition [%]
0 (control)	51.6 ± 3.7	3.7 ± 0.3	-
5	44.8 ± 4.8	3.5 ± 0.4	13
10	37.8 ± 4.6	3.2 ± 0.40	27
20	11.0 ± 0.9	1.5 ± 0.1	79
30	0.8 ± 0.8	-	98
50	0.0	-	100

**Table 2:** The effect of ammonia added at the start of cultivation on the growth of HLSCs over 120 h. Growth inhibition was defined as a reduction in cell density and population doublings of less than 70% of the control flask (0 mM ammonia). Data are means ± SD (n = 3).

Added Ammonia Concentration [mM]	Cell Density [×10^3^ Cells cm^−2^]	Population Doublings [−]	Growth Inhibition [%]
0 (control)	51.3 ± 3.4	3.7 ± 0.2	-
1	41.7 ± 7.4	3.4 ± 0.6	19
2	15.8 ± 2.3	2.0 ± 0.3	70
4	14.4 ± 2.0	1.9 ± 0.3	72
10	11.1 ± 8.5	1.5 ± 1.1	79
20	3.6 ± 8.5	-	93

**Table 3:** Growth characteristics of HLSCs cultured in the XPN10 bioreactor in αMEM growth medium at 37 °C and a 5% CO_2_ humidified atmosphere for 120 h. The XPN10 was inoculated with 4 × 10^3^ cells cm^−2^ and aerated with pure oxygen.

Run	Cell Density [×10^3^ Cells cm^−2^]	Cell Yield [×10^8^ Cells]	Population Doublings [−]	Doubling Time [h]
1	101	6.2	4.0	30
2	84	5.1	3.8	32
3	99	6.1	4.4	27
Mean ± SD	94 ± 8	5.8 ± 0.5	4.1 ± 0.3	30 ± 2

**Table 4:** Growth characteristics of HLSCs cultured in an STR using Cytodex 1 microcarriers in αMEM growth medium at 37 °C and a 5% CO_2_ humidified atmosphere for 120 h. The STR was inoculated with 4 × 10^3^ cells cm^−2^ and aerated with air.

Run	Cell Density [×10^3^ Cells cm^−2^]	Cell Yield [×10^8^ Cells]	Population Doublings [−]	Doubling Time [h]
1	12.7	1.7	1.9	39
2	31.8	4.2	3.9	25
3	25.4	3.3	3.6	30
Mean ± SD	23.3 ± 7.9	3.1 ± 1.0	3.1 ± 0.9	31 ± 6

## Data Availability

The authors declare that the data of this research are available from the corresponding authors upon reasonable request.

## Supplementary Materials

The following supporting information can be downloaded at https://www.mdpi.com/article/10.3390/bioengineering11070692/s1. Figure S1: Morphological observation of HLSCs during the attachment phase after (a) 1 h, (b) 2 h and (c) 3 h. Light spots indicate non-spreading, round cells. HLSCs were seeded at 4 × 10^3^ cells cm^−2^ and cultured in αMEM growth medium at 37 °C and a 5% CO_2_ humidified atmosphere; Figure S2: HLSC cell densities after 120 h in culture using different seeding densities. HLSCs were cultured in αMEM growth medium at 37 °C and a 5% CO_2_ humidified atmosphere. Data are means ± SD (n = 3); Figure S3: HLSC growth kinetics with (AB) and without antibiotics. HLSCs were seeded at 4 × 10^3^ cells cm^−2^ and cultured for 168 h at 37 °C in a 5% CO_2_ humidified atmosphere. Data are means ± SD (n = 3); Figure S4: Morphological observation of HLSCs during long-term experiment. (a) HLSCs with hLSC-like morphology after 28 cPDs (nine passages). (b) HLSCs with morphological abnormalities such as elongation and high granularity after 30.7 cPDs (10 passages); Figure S5: Microscopic observation of HLSCs stained with SYBR-Green on Cytodex 1 microcarriers after 120 h in culture in a STR (cell nuclei are shown in green). HLSCs were cultured in αMEM growth medium at 37 °C and a 5% CO_2_ humidified atmosphere. Scale bar = 1000 µm; Figure S6: Growth and metabolism of HLSCs on Cytodex 1 microcarriers in the STR (Run 3 as a representative of all runs). (a) Offline (hemocytometer) and online (permittivity) detection of HLSCs. (b) Glucose consumption and lactate production. After 120 h, all the glucose was consumed, necessitating supplementation. HLSCs were unable to recover from glucose starvation, resulting in significant cell loss; Table S1: List of antibodies used for CD marker determination; Table S2: Phenotyping of HLSCs during the long-term experiment. HLSC were seeded at 3 × 10^3^ cells cm^−2^ and cultured at 37 °C in a 5% CO_2_ humidified atmosphere; Table S3: HLSCs were harvested with diluted TrypLE after different incubation times to evaluate harvesting efficiency based on cell density, number and size of agglomerates. HLSCs were cultured in αMEM growth medium for 120 h at 37 °C and a 5% CO_2_ humidified atmosphere before harvesting; Table S4. Comparison of T-flask, Xpansion bioreactor and stirred-tank bioreactor as representatives of static, semi-dynamic and dynamic bioreactors, respectively. (−) No direct control/monitoring or no; (+) monitoring; (++) semi-controlled or limited; (+++) fully controlled or high.
