# Supplementary material for: Large-Scale Expansion of Human Liver Stem Cells Using Two Different Bioreactor Systems
Source: Bioengineering (Basel). 2024 Jul 9;11(7):692. doi: 10.3390/bioengineering11070692 (PMC11274206; doi:10.3390/bioengineering11070692)
Supplement: Supplementary file 1 [file bioengineering-11-00692-s001.zip › bioengineering-3091652-supplementary.pdf]

Table S1. List of antibodies used for CD marker determination.

Antibody	Manufacturer	Product number
CD44-PE	Miltenyi Biotec, Bergisch Gladbach, Germany	130-113-342
CD105-PE	Miltenyi Biotec	130-112-321
CD73-APC	BD Biosciences, Heidelberg, Germany	560847
CD90-FITC	Miltenyi Biotec	130-117-684
CD29-FITC	Miltenyi Biotec	130-123-692
CD14-PE	Miltenyi Biotec	130-110-577
CD34-FITC	BD Biosciences	555821
CD45	BD Biosciences	555482
Human albumin-FITC	LSBIO, Eching, Germany	LS–C68850
Human albumin-FITC- isotype	LSBIO	LS-C149360
PE-isotype	Miltenyi Biotec	130-113-200
FITC-isotype	Miltenyi Biotec	130-113-199
APC-isotype	Miltenyi Biotec	130-113-196

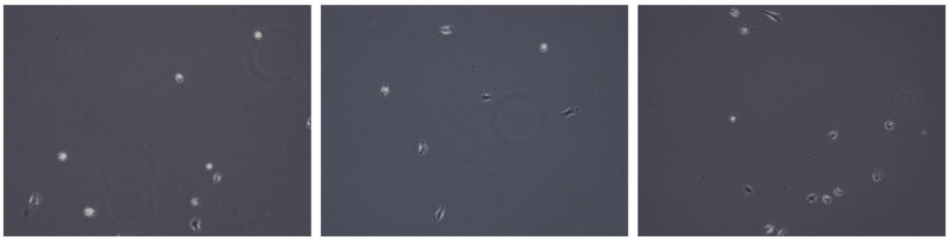

(a) (b) (c)

Figure S1. Morphological observation of HLSCs during the attachment phase after (a) 1 h, (b) 2 h and (c) 3 h. Light spots indicate non-spreading, round cells. HLSCs were seeded at 4 × 10^3 cells cm^{-2} and cultured in αMEM growth medium at 37 °C and a 5% CO_2 humidified atmosphere.

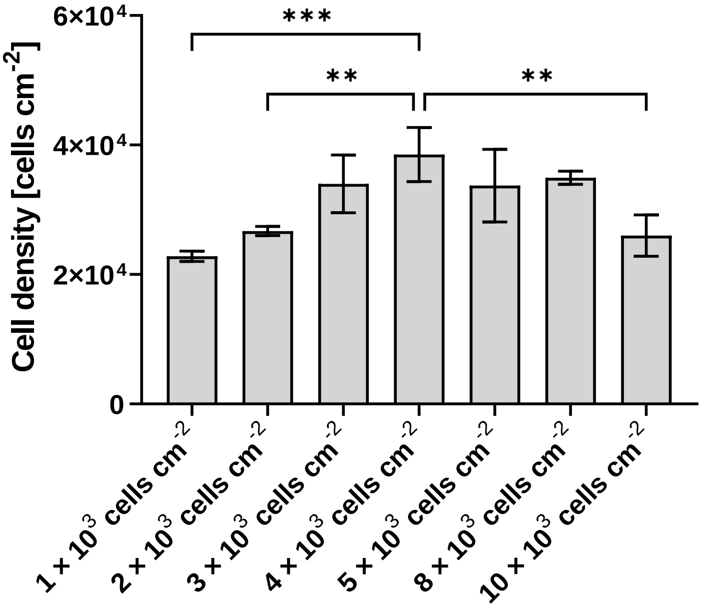

Figure S2. HLSC cell densities after 120 h in culture using different seeding densities. HLSCs were cultured in αMEM growth medium at 37 °C and a 5% CO_2 humidified atmosphere. Data are means ± SD (n = 3).

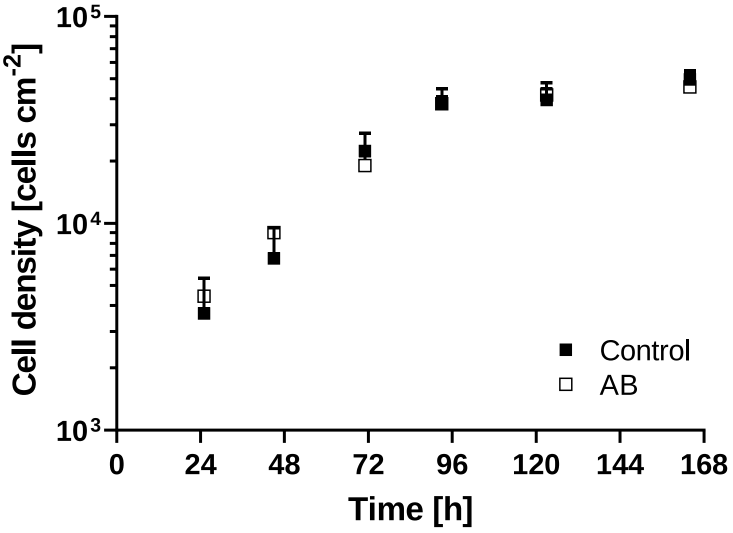

Figure S3. HLSC growth kinetics with (AB) and without antibiotics. HLSCs were seeded at 4 × 10^3 cells cm^{-2} and cultured for 168 h at 37 °C in a 5% CO_2 humidified atmosphere. Data are means ± SD (n = 3).

Table S2. Phenotyping of HLSCs during the long-term experiment. HLSC were seeded at 3 × 10^3 cells cm^{-2} and cultured at 37 °C in a 5% CO_2 humidified atmosphere.

Cumulative population doublings, cPDs [-]	CD29 [%]	CD73 [%]	CD105 [%]
21	100	100	99
28	100	100	99
30.7	98	100	96
33.7	100	100	82
35.3	99	100	84

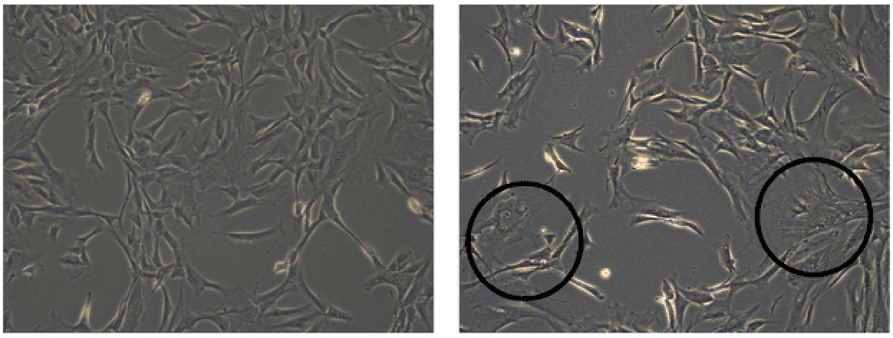

(a) (b)

Figure S4. Morphological observation of HLSCs during long-term experiment. (a) HLSCs with hLSC-like morphology after 28 cPDs (nine passages). (b) HLSCs with morphological abnormalities such as elongation and high granularity after 30.7 cPDs (10 passages).

Table S3. HLSCs were harvested with diluted TrypLE after different incubation times to evaluate harvesting efficiency based on cell density, number and size of agglomerates. HLSCs were cultured in αMEM growth medium for 120 h at 37 °C and a 5% CO_2 humidified atmosphere before harvesting.

TrypLE concentration [%]	Incubation time [min]	Cell density after harvest [× 10^3 cells cm^{-2}]	Agglomerate number [-]	Agglomerates size [cells per agglomerate]
100	5	54	0	< 5
100	15	68	0	< 5
50	20	60	1	5–10
25	20	45	3	10–15
12.5	20	44	1	15–20

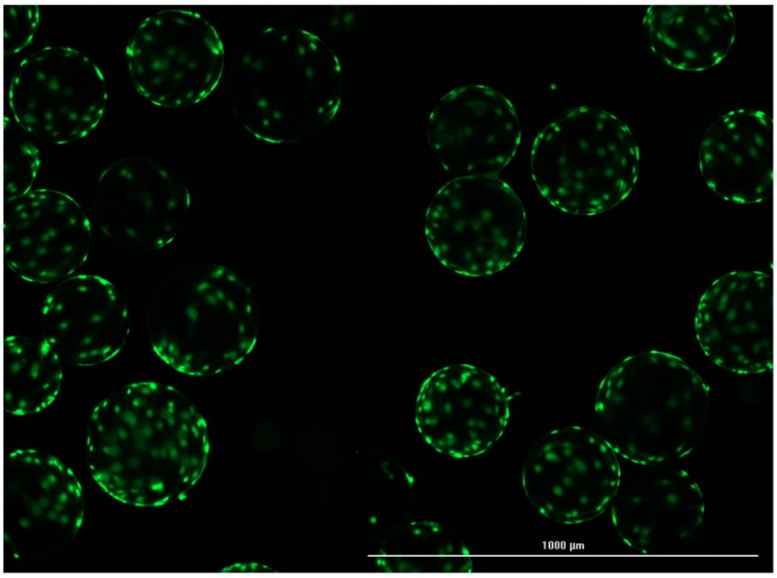

Figure S5. Microscopic observation of HLSCs stained with SYBR-Green on Cytodex 1 microcarriers after 120 h in culture in a STR (cell nuclei are shown in green). HLSCs were cultured in αMEM growth medium at 37 °C and a 5% CO_2 humidified atmosphere. Scale bar = 1000 µm.

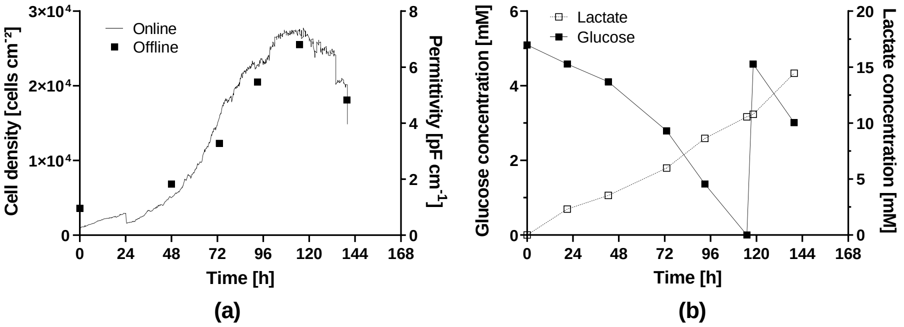

Figure S6. Growth and metabolism of HLSCs on Cytodex 1 microcarriers in the STR (Run 3 as a representative of all runs). (a) Offline (hemocytometer) and online (permittivity) detection of HLSCs. (b) Glucose consumption and lactate production. After 120 h, all the glucose was consumed, necessitating supplementation. HLSCs were unable to recover from glucose starvation, resulting in significant cell loss.

Table S4. Comparison of T-flask, Xpansion bioreactor and stirred-tank bioreactor as representatives of static, semi-dynamic and dynamic bioreactors, respectively. (-) No direct control/monitoring or no; (+) Monitoring; (++) Semi-controlled or limited; (+++) Fully controlled or high.

**Parameter / Criterion**	**T-flasks (static)**	**Xpansion Bioreactor (semi-dynamic)**	**Stirred-tank Bioreactor (dynamic)**
Temperature	-	+	+++
pH	-	++	+++
Dissolved oxygen (DO)	-	++	+++
Biomass	+*	+*	+++
Metabolites	-	-	+++
Manual Effort	Large	Medium	Low
Automatization	-	++	+++
Scalability	-	++	+++

* Biomass may be estimated by microscopical observations
